# Adenosine Receptors in Neuropsychiatric Disorders: Fine Regulators of Neurotransmission and Potential Therapeutic Targets

**DOI:** 10.3390/ijms23031219

**Published:** 2022-01-22

**Authors:** Silvia Pasquini, Chiara Contri, Stefania Merighi, Stefania Gessi, Pier Andrea Borea, Katia Varani, Fabrizio Vincenzi

**Affiliations:** 1 Department of Translational Medicine, University of Ferrara, 44121 Ferrara, Italy; psqslv@unife.it (S.P.); chiara.contri@unife.it (C.C.); mhs@unife.it (S.M.); gss@unife.it (S.G.); fabrizio.vincenzi@unife.it (F.V.); 2 University of Ferrara, 44121 Ferrara, Italy; bpa@unife.it

**Keywords:** adenosine, neurotransmission, adenosine receptors, neuropsychiatric disorders, mood disorders, neurodevelopment disorders

## Abstract

Adenosine exerts an important role in the modulation of central nervous system (CNS) activity. Through the interaction with four G-protein coupled receptor (GPCR) subtypes, adenosine subtly regulates neurotransmission, interfering with the dopaminergic, glutamatergic, noradrenergic, serotoninergic, and endocannabinoid systems. The inhibitory and facilitating actions of adenosine on neurotransmission are mainly mediated by A_1_ and A_2A_ adenosine receptors (ARs), respectively. Given their role in the CNS, ARs are promising therapeutic targets for neuropsychiatric disorders where altered neurotransmission represents the most likely etiological hypothesis. Activating or blocking ARs with specific pharmacological agents could therefore restore the balance of altered neurotransmitter systems, providing the rationale for the potential treatment of these highly debilitating conditions. In this review, we summarize and discuss the most relevant studies concerning AR modulation in psychotic and mood disorders such as schizophrenia, bipolar disorders, depression, and anxiety, as well as neurodevelopment disorders such as autism spectrum disorder (ASD), fragile X syndrome (FXS), attention-deficit hyperactivity disorder (ADHD), and neuropsychiatric aspects of neurodegenerative disorders.

## 1. Introduction

The endogenous autacoid adenosine is found in all mammalian tissues, where it plays an important role: it is the major constituent of ATP and it regulates a variety of physiological functions, but it also has a key role in many pathologies such as cancer, as well as in inflammatory and neurological diseases. In the central nervous system (CNS), adenosine controls neuronal excitability, synaptic plasticity, and neuron degeneration. It is even involved in astrocytic and microglial cell modulation [1]. The major mechanism underlying adenosine production is the dephosphorylation of the adenine nucleotides (ATP, ADP, and AMP) [2]. Physiologically, a part of ATP is dephosphorylated to adenosine. In the case of cellular stress such as injury, hypoxia, neurodegeneration, neuroinflammation, or excitotoxicity, the rate of adenosine production is enhanced. Adenine nucleotides are more frequently released extracellularly. Here, ectoenzymes located on the cell membrane called ecto-5′nucleotidase (CD73) and ecto-nucleoside triphosphate phosphohydrolase (CD39) dephosphorylate them into adenosine. Extracellular adenosine is then degraded to inosine by the adenosine deaminase enzyme (ADA). In humans, adenosine could be further degraded to uric acid or be re-uptaken in cells by specific nucleoside transporters (ENT1 and ENT2) and phosphorylated again to ATP [3]. Adenosine can arise its effects through the interaction with different adenosine receptors (ARs): A_1_, A_2A_, A_2B_, and A_3_ARs. They are components of the G-protein-coupled receptor (GPCR) family and bear the typical structure of membrane receptors characterized by seven transmembrane domains. ARs are able to start distinct signal transduction pathways thanks to their link to different G-proteins [2,4]. In normal conditions, adenosine has a higher affinity for A_1_ARs and A_2A_ARs, but when its concentration arises, as in pathological status, it is able to also activate the lower affinity A_2B_ARs and A_3_ARs [3].

The two AR subtypes most expressed in the CNS are A_1_ARs and A_2A_ARs. The latter is present in almost all the districts, with its major expression in the striatum [5]. Pre- and postsynaptic neurons, astrocytes, microglia, oligodendrocytes, and capillary endothelial cells all contain A_2A_ARs [6,7]. After stimulation, A_2A_ARs couple to G_s_, or G_olf_ in the striatum, and activate adenylate cyclase, heightening cAMP concentration and stimulating protein kinase A (PKA) and many downward targets or other signaling pathways [1,8]. A_2A_ARs in the brain are involved in the control of voluntary movements and in motivational, emotional, and cognitive processes [8]. Homomeric A_2A_ARs but also their capability to form heteromers with other receptors have an important role in these processes. Interactions between A_2A_ARs and dopamine D_2_ receptors, metabotropic glutamate type 5 receptors (mGlu5R), the cannabinoid CB_1_ receptor, and other AR subtypes have been identified [9,10]. Brain-localized A_1_ARs have been found in pre- and postsynaptic neurons. In the first case, they impede glutamate, dopamine, serotonin, and acetylcholine release. At the postsynaptic level, A_1_ARs impair neuronal signaling through hyperpolarizing neuron membrane and reducing excitability via potassium channel modulation. A_1_ARs can form heteromers with A_2A_ARs and with dopamine D_1_ receptors, promoting self-adaptive changes regulating neural plasticity [11,12].

Given the intricate network of effects and interactions of ARs in the brain (Table 1), this review focuses on their role in neuropsychiatric aspects of CNS disorders including psychotic and mood disorders, as well as neurodevelopmental and neurodegenerative pathologies.

## 2. Adenosine

### 2.1. Psychotic and Mood Disorders

#### 2.1.1. Schizophrenia

Schizophrenia is a serious psychiatric disease that affects 1% of the global population. Typical symptoms of schizophrenia are hallucinations, delusions (the most common being persecutory delusion), social avoidance, lack of vitality and attention, and working memory deficits. Unfortunately, the etiology of this pathology is still largely unknown [13,14]. The most accredited theory explaining schizophrenia postulates the hyperfunction of the mesocorticolimbic dopamine system and the glutamate system hypofunctionality [15,16]. The hypothesis involving the dopamine system is supported by experimental evidence reporting, in limbic structures of schizophrenic patients, a hyperdopaminergic state, with the dopamine concentration related to symptom intensity, and a major amount of dopamine D_2_ receptors bounded by endogenous dopamine: psychotic manifestations seem to be accountable to these alterations [17]. Nevertheless, drugs blocking dopamine receptors do not show good efficacy for the treatment of schizophrenia; moreover, their use is connected to many adverse effects and drug resistance [5].

Different studies, both in animals and in humans, reported that N-methyl-D-aspartate (NMDA) receptor antagonist treatment causes schizophrenia-like psychotic and cognitive symptoms [18]. These observations generate another hypothesis, which could explain all schizophrenic symptoms on the basis of the hypofunctionality of the glutamatergic system without excluding dopaminergic modifications [19]. In fact, NMDA receptor antagonists are known to cause dopamine release, thus hyperactivating the dopaminergic system [20]. Moreover, the chronic administration of NMDA antagonists modifies dopaminergic transmission, causing alterations analogous to schizophrenic ones [21]. Unfortunately, the potential excitotoxic effects due to NMDA receptor activation make the treatment with NDMA agonists not pursuable. The most effective approach could be the simultaneous normalization of dopaminergic and glutamatergic systems. All things considered, the modulation of the adenosine pathway should represent an encouraging strategy for schizophrenia treatment [22,23].

Adenosine, as mentioned above, is a neuromodulator involved in controlling information flood in neuron networks through the activation of inhibitory A_1_ARs and facilitatory A_2A_ARs, the two most abundant AR subtypes in the brain [5]. Adenosine is also involved in glutamatergic and dopaminergic modulation [24].

**Figure 1:**
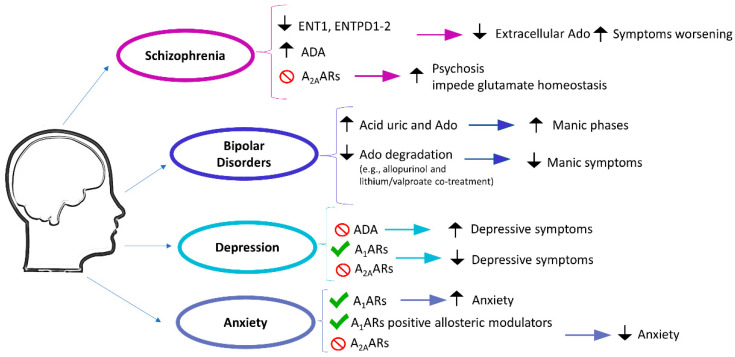
Involvement of adenosinergic system in psychotic and mood disorders. ENT1, equilibrative nucleoside transporter 1; ENTPD, ectonucleoside triphosphate diphosphohydrolase; ADA, adenosine deaminase. Green check mark, activation; red prohibition sign, blockade; upward arrow, increase; downward arrow, decrease.

In an animal model of transgenic mice that overexpress adenosine kinase, researchers observed the development of cognitive and locomotor impairments similar to those found in schizophrenia [23]. This led to the hypothesis that adenosine hypofunction causes changes in dopaminergic and glutamatergic signaling (Figure 1) [22,23]. Accordingly, the hyperdopaminergic condition, due to decreased inhibition of the dopamine pathway and the deregulation of the glutamate pathway mediated by A_2A_ARs, is probably related to the decrease of extracellular adenosine, maybe also through the involvement of A_1_ARs [5]. In this, A_2A_ARs stimulation antagonizes psychotomimetic-induced motor activity, while the AR antagonist caffeine exacerbates psychosis in schizophrenic patients [25].

On the contrary, a later paper showed that the lower extracellular adenosine levels, which are posited to be partly responsible for glutamatergic and dopaminergic system dysregulation and schizophrenia manifestations, seem not to be related to adenosine kinase overexpression [22,26,27]. In fact, emerging evidence suggests that, in schizophrenia, the minor availability of extracellular adenosine may be caused by an alteration in other pathways of adenosine metabolism. The expression CD39, which converts ATP to ADP and AMP, is reduced in the schizophrenic dorsolateral prefrontal cortex astrocytes, resulting in reduced production of AMP, the substrate for adenosine. Moreover, increased ADA expression and reduced levels of ENT1 in enriched populations of pyramidal neurons in schizophrenia have been found [28]. The reduction of CD39 activity in the schizophrenic striatum was also confirmed by other studies [29]. Several pieces of evidence suggest that purinergic signaling may be a target of antipsychotic drugs. It is worth noting that chronic treatment with the antipsychotic clozapine increased the activity of striatal 5′-nucleotidase (CD73) in rats, an effect not observed with haloperidol [30]. However, haloperidol, as well as olanzapine and sulpiride, inhibited CD39 activity in zebrafish brain membranes, without affecting CD73 activity [31]. In a subsequent work performed in zebrafish brain, the same authors confirmed that haloperidol inhibited CD39 and ADA gene expression [32]. In humans, while schizophrenic patients treated with clozapine showed increased serum adenosine deaminase, no differences were found on CD73 activity [33].

The involvement of ARs in schizophrenia is testified by many preclinical studies [5]. A_2A_AR stimulation and dopaminergic blockade have been demonstrated to provoke analogous behavioral effects and to prevent the motor-exciting effects induced by amphetamine or dopamine agonist treatment [25,34,35]. Consequently, A_2A_ARs are regarded as atypical antipsychotic drugs that provide their effects through the interaction with A_2A_–D_2_ heteromers, wherein they inhibit D_2_-mediated G_i/o_ signaling and increase the D_2_-mediated β-arrestin 2 pathway [36,37,38]. Even the activation of A_2A_ARs homodimers has antipsychotic effects that are mediated by the G–adenylate cyclase–PKA pathway and the subsequent increase of the striatopallidal γ-aminobutyric acid (GABA) pathway thanks to α-amino-3-hydroxy-5-methyl-4-isoxazolepropionic acid (AMPA) and NMDA receptor phosphorylation [39].

In schizophrenia, the glutamatergic dysfunction theory also involves the glial glutamate transporter-I (GLT-I) [40]. In this framework, A_2A_ARs seem to be very important. A_2A_AR knock-out astrocytes show an exaggerated GLT-1 activity, impeding glutamate homeostasis and leading to psychomotor and cognitive disability. Taken together, these data suggest that astrocytes may play a crucial part in schizophrenia pathophysiology [41].

Even though the therapeutic use of A_2A_AR agonists has been hampered by their cardiovascular adverse effects, adenosine-increasing drugs, such as allopurinol and dipyridamole, have been used in schizophrenia. Using adenosine concentration modulators may be a promising therapeutic strategy since adenosine generating enzymes and transporters appear to be deregulated in patients with schizophrenia [5]. Nonetheless, the effectiveness of this therapeutic approach needs to be more deeply investigated.

#### 2.1.2. Bipolar Disorders

Bipolar disorders are defined as persistent, recurring diseases affecting more than 1% of the global population. They represent one of the major causes of disability in youngsters, provoking cognitive and functional disabilities and augmented mortality, in particular, due to suicide and cardiovascular diseases. Bipolar patients often suffer from psychiatric and non-psychiatric concurrent pathologies, which may increase mortality. Bipolar disorders are mostly heritable; nonetheless, their etiology is probably due to both genetic and environmental factors [42,43].

The first proof of AR participation in bipolar disorders is based on the major release of uric acid, a product of adenosine metabolism, in manic subjects (Figure 1). Following studies have reinforced the idea that bipolar disorders, in particular manic phases, are characterized by a dysfunction of the purinergic system [44,45,46,47]. A positive effect of the co-treatment with allopurinol, which heightens adenosine concentration through the inhibition of purine degradation, and lithium or valproate has been observed in bipolar patients with manic symptoms [48,49]. Interestingly, allopurinol was not effective in the absence of lithium or valproate [47,50].

However, it is not yet clear as to whether these findings indicate adenosine dysfunction in bipolar disorder in the brain [51,52]. The specific involvement of A_1_ARs has still to be clarified; until now, data suggest that A_1_ARs are upregulated by sleep deprivation with antidepressant effects but also triggering maniac symptoms in bipolar patients [53]. Other studies highlight that carbamazepine, a drug used in acute and dysphoric mania, can also act as an A_1_ARs antagonist [54].

#### 2.1.3. Depression

Depression is a mood disorder characterized by persevering sorrow and lack of heed. It is classified as major depression, persistent depression (dysthymia), premenstrual dysphoric disorder, and depressive disorder due to other pathologies. All kinds of depressive disorders share ordinary characteristics such as sorrow, desolation, or irritable mood, followed by somatic and cognitive changes that seriously influence an individual’s life [55]. Depression neurobiological basis is still ineffectively characterized, and albeit the norepinephrine and serotonin insufficiency hypothesis is the most acknowledged; however, new investigations have shown that different intracellular pathways involved in neuroplasticity might be answerable for this disorder [5]. Even the adenosinergic system seems to be involved in anxiety, the AR stimulation, or the inhibition of ADA, which enhances adenosine levels, resulting in depressive behavior. On the other hand, adenosine shows antidepressant effects (Figure 1). Sleep deprivation, which enhances adenosine levels and upregulates A_1_ARs, could be an alternative strategy to treat drug-resistant patients [56,57].

A_2A_AR capability to modulate synaptic activity and their elevated expression in mesolimbic pathways, which are implicated in motivational behaviors, encourage exploiting this receptor subtype as a new target in depressive disorder treatment. In fact, they were the first AR subtype found to be involved in depression; in particular, A_2A_AR antagonists show antidepressant effects [54]. Later, in transgenic rats, an overexpression of A_2A_ARs in forebrain neurons was discovered to be linked to enhanced depression-like behavior and anhedonia, one of the principal characteristics of depression [58]. Chronic mild stress conditions in rodents appear to lead to depressive-like behavior and to be associated with reduced synaptic plasticity and synaptic protein density and increased A_2A_ARs in the striatum and hippocampal glutamatergic terminals [59,60]. An A_2A_AR augment intercedes for the synaptic and behavioral changes due to prolonged stress conditions. In fact, these effects are reverted by treatment with caffeine, selective A_2A_AR antagonists, or by genetic deletion of A_2A_ARs in forebrain neurons [60]. A recent study performed in mice reports that treatment with DMPX, a selective A_2A_AR antagonist, augments the effect of antidepressant drugs such as tianeptine and agomelatine [61]. Moreover, A_2A_AR antagonism is able to revert deficits induced by stress due to maternal separation in rats [62]. The thus-far antidepressant effect of A_2A_AR blockade seems to be contradictory with the data reporting a brain-derived neurotrophic factor (BDNF) expression upregulation induced by A_2A_AR agonism in rat primary neurons, particularly since BNDF is known for its antidepressant effects [63,64]. A_2A_AR stimulation effects on BDNF seem to be convoluted. In the hippocampus, A_2A_ARs influence BDNF effects on GABAergic transmission, altering glutamatergic inputs to pyramidal neurons and cholinergic inputs to GABA interneurons [65]. Moreover, BDNF seems to be linked to both antidepressive and prodepressive behaviors, depending on the cerebral area and the cells involved [64]. The mechanism underlying the antidepressant effect of A_2A_AR antagonists is still unclear, but one likely theory is the interaction of A_2A_ARs with A_1_ARs. Since A_2A_ARs generally impede A_1_AR actions, the blockade of A_2A_ARs could result in the facilitated activity of A_1_ARs [66].

In particular, A_1_AR activation provokes antidepressant effects in transgenic mice in which the overexpression of A_1_ARs could be turned on or off [56]. In this mouse model, A_1_ARs expressed on neurons are responsible for the antidepressant effect because A_1_ARs transgene expression is limited to calcium/calmodulin-dependent protein kinase type II forebrain neurons [56,67]. A_1_AR upregulation, activating the transgene, leads to a marked resistance to depressive behavior. Conversely, in A_1_AR knock-out mice, an enhanced depressive behavior and a resistance to the antidepressant effects of sleep deprivation were observed, suggesting sleep deprivation effects are mostly due to A_1_AR upregulation [56]. It has also been demonstrated that the A_1_AR antidepressant effect is related to the immediate early gene Homer1a, a gene increased by many antidepressant treatments such as sleep deprivation; imipramine; ketamine; and, of course, A_1_AR activation. In this context, it has been reported that small interfering ribonucleic acid knockdown of Homer1a enhances depressive-like behavior and prevents the antidepressant effects of A_1_AR upregulation. Consequently, Homer1a, in the medial prefrontal cortex, represents a shared signaling pathway that mediates the antidepressant effects of both A_1_AR stimulation and many antidepressant drugs [56,68]. A recent study reports that the Homer1a activation effect is due to its activation of mGluR5, which enhances AMPA receptor-mediated synaptic transmission [69].

#### 2.1.4. Anxiety

Generalized anxiety represents one of the prevailing mental disorders, affecting up to 20% of adults each year. It is characterized by fright, concern, and a continual feeling of being overcome with a persevering, uncontrolled, and unreasonable worry about daily activity. The concern may regard financial, familiar, healthy, and future aspects. Excessive worry is the principal feature of anxiety disorder—it is hard to handle, and frequently goes along with different nonspecific psychological and physical manifestations [70,71]. Anxiety disorders comprise various mental illnesses that can be divided into classic phobias, social phobias, obsessive–compulsive disorder, and panic attack—the incidence of these conditions is twice as high in women as in men [72]. The gold standard therapy is represented by selective serotonin reuptake inhibitors and benzodiazepines; nonetheless, the prolonged use may lead to different adverse effects [73].

Adenosine involvement in anxiety has firstly been highlighted by coffee consumption. Although it is well established that elevated caffeine intake can provoke anxiety, it is also recognized that caffeine consumption consequences depend on the quantity and the coffee-drinking habits, the subject’s susceptibility to anxiety, and the presence of concomitant stress conditions and associated changes in the hypothalamic–pituitary–adrenal axis [74]. Studies in various anxiety animal models demonstrate that acute treatment with non-selective A_1_AR and A_2A_AR antagonists leads to anxiogenic effects (Figure 1). Conversely, adenosine or adenosine increasing molecules, for instance, ENT1 inhibitor, or in ENT1 knock-out mice, produce poor anxiety levels [58,75]. In post-traumatic stress, WS0701, an adenosine derivative, is able to decrease fear and anxiety [76]. The genetic deletion of A_1_ARs or A_2A_ARs in mice provokes anxiogenic behavior; unfortunately, the selective blockade of these receptor subtypes did not explain which one receptor is involved [74]. Moreover, in the striatum, A_2A_ARs deletion does not modify anxiety-like behavior, despite the fact that if the deletion comprises the cortex and hippocampus, mice will exhibit an anxiolytic behavior [77]. These results are conflictual with those showing the anxiogenic role of caffeine and the connection between the A_2A_AR gene and panic disorder [78]. Conversely, adenosine treatment in mice causes an anxious behavior not present in A_2A_AR knock-out mice. These effects probably depend on the activation of caspase-1 and the enhanced IL-1β release caused by A_2A_ARs located in the amygdala [79]. However, the therapeutic potential of A_2A_ARs has not been fully clarified. A_2A_AR stimulation seems to cause anxiolytic or null effects, while their blockade does not have any effect on anxiety. Recently, it has been reported that in a prolonged stress rat model, A_2A_AR antagonist long-term treatment improves gender-specific microglial modifications in the prefrontal cortex, together with anxiety-like behavior in males, but not in females [80]. To date, modulation of A_2A_ARs is not deemed as a potential anxiolytic treatment, both because of the limitations of the animal models on which studies are conducted and because of the low expression of these receptors in the areas of the brain involved in anxiety.

An encouraging target for the management of anxiety is represented by the A_1_AR subtype since its stimulation can regulate neuronal activity through neurotransmitter release blockade [81]. A_1_AR upregulation in forebrain neurons elicits antidepressant effects [56]. Many lines of evidence demonstrate that A_1_AR knock-out mice show increased anxiety [82,83,84]. Although they have promising therapeutic potential, the exploitation of A_1_AR agonists is impeded by important adverse effects and low selectivity [85]. Specifically, A_1_AR stimulation provokes negative chronotropic and inotropic effects in the heart, catalepsy, and locomotor activity depression [81,86]. Considering this background, positive allosteric modulation represents an attractive option instead of orthosteric ligands. Allosteric enhancers, binding to a different site and enhancing the endogenous agonist effects, display a minor side effect profile than orthosteric agonists, lower receptor desensitization, and a higher receptor subtype selectivity [87,88]. In the last decade, different series of A_1_AR-positive allosteric modulators have been developed and characterized [89,90]. One of the most potent A_1_AR-positive allosteric modulators synthesized up to this point, TRR 469, exhibited strong anxiolytic effects similar to those of diazepam. Moreover, in mouse brain membranes, TRR 469 enhances affinity of CCPA, an A_1_AR agonist [4]. This is very promising considering the fact that the substantial advantage of positive allosteric modulators is the capability to augment endogenous agonist affinity, amplifying receptor activation in a more physiological manner [91].

### 2.2. Neurodevelopmental Disorders

#### 2.2.1. Autism Spectrum Disorder (ASD)

Autism spectrum disorder is a term used to describe a heterogeneous group of neurodevelopment disorders characterized by social communication deficits and repetitive, stereotyped behaviors [92]. ASD has been associated with altered brain development and neural reorganization linked to a plethora of genetic and environmental risk factors. A generally accepted aberration in ASD is the long-distance cortical and subcortical underconnectivity with short distance overconnectivity [93]. The current treatment options for ASD include pharmacological and non-pharmacological interventions. Current pharmacological therapeutic options for ASD include psychostimulants, atypical antipsychotic drugs, mood stabilizers, cholinesterase inhibitors, alpha-2 adrenergic receptor agonists, antidepressants, and NMDA receptor antagonists [94].

Several experimental pieces of evidence point to adenosine’s involvement in ASD, indicating its receptors as potential pharmacological targets for ASD treatment. In a randomized, double-blind, placebo-controlled clinical trial involving 48 children with ASD treated with risperidone, the adenosine reuptake blocker and xanthine phosphodiesterase inhibitor propentofylline was evaluated as an adjunctive treatment. Children receiving propentofylline had a better score in the Childhood Autism Rating Scale (CARS) compared with the placebo group [95]. Using a customized parent-based questionnaire, Masino and co-workers highlighted an improvement in behavioral symptoms in children with ASD following activities expected to increase adenosine levels [96].

Among the AR subtypes, A_2A_ARs have been identified as the ones most implicated in ASD, although activation of A_1_ARs is also implicated in the reduction of some symptoms related to ASD. The association between single-nucleotide polymorphisms (SNPs) in the A_2A_AR gene and ASD has been studied; a nominal association with the disorder was observed for rs2236624-CC, while rs3761422, rs5751876, and rs35320474 affected phenotypic variability in ASD symptoms [97].

One of the earliest pieces of evidence has been obtained with the A_2A_AR agonist CGS 21680 and the non-selective AR agonist NECA, which attenuated amphetamine-induced stereotypy in rats, while the A_2A_AR antagonist DMPX potentiated stereotypy [98].

The C58 mouse strain represents a useful model for the aberrant repetitive behavior characteristic of a number of neurodevelopmental disorders, including ASD [99]. In C58 mice, the administration of the A_1_AR agonist CPA or the A_2A_AR agonist CGS 21680 did not reduce repetitive behavior. However, when the two agonists were used in combination, a significant reduction in repetitive behavior was observed [100]. This effect was accompanied by an increase in Fos transcription in the dorsal striatum, wherein Fos transcription was used as an index of neuronal activity in both direct and indirect pathway neurons [100]. The reduction of repetitive behavior with the co-administration of CGS 21680 and CPA was previously reported in deer mice, which develop high levels of repetitive motor behaviors when reared in a standard laboratory environment [101].

In the BTBR T+ *Itpr3^tf^*/J (BTBR) mouse model of idiopathic autism, the acute administration of the A_2A_AR agonist CGS 21680 reduced the self-grooming behavior as well as learning deficits evaluated using a spatial reversal learning test [102]. The therapeutic potential of A_2A_AR activation in ASD appears to be linked not only to altered indirect basal ganglia pathway activity, but also to an imbalance in the production of pro- and anti-inflammatory cytokines and transcription factors [103,104]. Several papers highlighted that A_2A_AR activation with CGS 21680 could improve neuroimmune dysfunctions in BTBR mice, while administration of the A_2A_AR antagonist SCH 58261 exacerbated these dysfunctions [105,106,107,108,109].

#### 2.2.2. Fragile X Syndrome (FXS)

FXS is one of the most common forms of inherited intellectual disability and is caused by an expansion of CGG-repeats in the fragile X mental retardation 1 gene (*FMR1*), resulting in the loss of its product, fragile X mental retardation 1 protein (FMRP) [106]. FMRP is an RNA-binding protein involved in different steps of RNA metabolism and has a pivotal role in gene expression, regulating the synthesis of several proteins involved in neuronal synaptic connections [110]. Among the defects correlated with FRMP absence, one of the key consequences is the excessive glutamatergic signaling mediated by mGluR5. This results in increased long-term depression and augmented protein synthesis [111]. The blockade of mGluR5 signaling represents an encouraging approach for the pharmacological treatment of FXS and different agents are currently in development [112].

Adenosine, acting on A_2A_ARs, exerts a permissive role in the mGluR5-mediated effect, and A_2A_AR antagonists could therefore represent an interesting option for indirectly blocking mGluR5 overactivation in FXS. A recent study evaluated the role of A_2A_ARs in FXS by studying their interaction with mGlu5 receptors in an experimental model represented by *Fmr1* KO mice [113]. In hippocampal slices of *Fmr1* KO mice, the A_2A_AR antagonist ZM241385 inhibited the mGlu5R-induced depression of field excitatory postsynaptic potential (fEPSP) slope, while it was potentiated by the A_2A_AR agonist CGS21680. When compared to WT mice, *Fmr1* KO mice exhibit abnormally increased mGluR-dependent long-term depression (LTD). Interestingly, the treatment of *Fmr1* KO mice with istradefylline, an A_2A_AR antagonist, restored mGluR-dependent LTD to WT levels [113]. Istradefylline also reduced dendritic spine density; improved learning deficit; and decreased the expression of overactive phenotype markers in *Fmr1* KO mice, such as mammalian target of rapamycin (mTOR), tropomyosin receptor kinase B (TrkB), and striatal-enriched protein tyrosine phosphatase (STEP).

#### 2.2.3. Attention-Deficit Hyperactivity Disorder (ADHD)

ADHD is a debilitating neuropsychiatric condition characterized by high and persistent levels of overactivity, impulsivity, and inattention [114]. It is widely acknowledged that both environmental and genetic factors play a key role in ADHD [115]. Among the neurotransmitters, several pieces of evidence indicate a central role for dopamine in the pathogenesis of ADHD. Dopaminergic projections from the midbrain are thought to be involved in reinforcement learning mechanisms [116], and some of the symptoms of ADHD are correlated to alterations in dopamine functions [114]. Furthermore, the most commonly used pharmacological agent for ADHD, methylphenidate, increases extracellular dopamine levels by blocking the dopamine transporters (DAT) in the synapse [117]. As a matter of fact, some brain regions activated by dopamine are altered, as highlighted by imaging studies. Lastly, a significant association of ADHD with variants of DAT and dopamine receptor genes was found [118].

Considerable evidence indicates that dopamine interacts with adenosine in different brain areas. Several studies have reported that there are cellular interactions between dopamine D_1_ and D_2_ receptors and A_1_ and A_2A_ARs that are colocalized on the same basal ganglia neurons, which include the ability to form heteromeric complexes [119]. Functional studies showed that these receptor complexes are responsible for the antagonistic interactions between ARs and dopamine receptors, wherein activation of ARs dampens dopamine signaling. For these reasons, ARs have been considered as potential therapeutic targets for pathological conditions characterized by an imbalance of dopaminergic neurotransmission, including ADHD.

A study of the possible association between A_2A_AR gene polymorphisms and ADHD highlighted a nominal association between ADHD traits and three SNPs; for one of these, rs35320474, results remained significant after correction for multiple comparisons, indicating the possible involvement of the A_2A_AR gene in ADHD [120]. Anxiety is a common comorbidity with ADHD. It has been found that an interaction between A_2A_AR and dopamine D_2_ genes increases the risk of anxiety disorders in children with ADHD [121].

The spontaneously hypertensive rat (SHR) is considered a good experimental model for ADHD, as it displays hyperactivity, impulsivity, and reduced attention in different behavioral tasks [122]. Treatment with the non-selective AR antagonist caffeine, the A_1_AR antagonist DPCPX, the A_2A_AR antagonist ZM241385, or their combination improved the performance of SHR in the object-recognition task [123]. In another study, chronic caffeine treatment normalized dopaminergic function and improved memory and attention deficits in SHR [124]. Furthermore, an upregulation of A_2A_ARs was found in frontocortical nerve terminals in SHR. In a more recent study, the interaction between the cannabinoid and the adenosine systems was evaluated on impulsive behavior in SHR. It was found that the administration of the cannabinoid receptor agonist WIN55212-2 increased impulsive behavior. Surprisingly, an acute pre-treatment with the non-selective AR antagonist caffeine abolished the effects of WIN55212-2, whereas a chronic caffeine treatment increased impulsivity in SHR [125].

### 2.3. Neuropsychiatric Aspects in Neurodegenerative Diseases

#### 2.3.1. Parkinson’s Disease (PD)

PD is one of the most prevalent progressive neurodegenerative pathologies worldwide. The current therapeutic strategy addresses motor symptoms by acting prevalently on the dopaminergic pathway, which is well known to be altered in the disease [126]. Nevertheless, there are aspects of the PD condition that are underestimated, namely, the non-motor symptoms. These include depression and anxiety, sleep disorders, and cognitive dysfunctions, particularly memory problems. Non-motor symptoms are found at all stages of the disease and are one of the main causes of patients’ poor quality of life [127]. Non-motor symptoms can be difficult to diagnose and treat. Furthermore, current dopamine-based treatment strategies, such as levodopa, frequently have no effect on non-motor symptoms or may even worsen them [126,128]. Therefore, non-motor symptoms and their treatment are crucial issues in the treatment of Parkinson’s disease, with a strong impact on the quality of life of patients and caregivers [129]. Studies in PD patients have shown that the age of the patients, the duration and severity of the disease, and the dose and duration of levodopa therapy correlate positively with the degree of non-motor symptoms [130]. PD is generally associated with the loss of dopaminergic neurons, but numerous other neurotransmitter systems are involved in the pathogenesis and progression of the disease, and these mechanisms may be behind the appearance of non-motor symptoms. Of particular importance among these are the GABA, glutamate, serotonin, noradrenaline, and acetylcholine systems. Furthermore, it is important to mention the heteromerization of dopamine receptors D_2_ with A_2A_ARs, which is one of the most studied receptor interactions [131,132]. In PD, A_2A_ARs play an extremely valuable role in movement control as their expression is predominant in the striatum. Their presence is not limited to the striatum—they are also found in limbic areas, the nucleus accumbens, the amygdala, the hippocampus, the hypothalamus, the thalamus, the frontal cortex, and the cerebellum, implying that A_2A_ARs play a role in non-motor symptoms [133].

Depression is the most common neuropsychiatric symptom associated with PD. It is mainly characterized by a loss of interest and pleasure, differentiating it from other types of depression. Studies have shown that dopaminergic therapy increases depression in patients [134]. Istradefylline, an A_2A_ARs antagonist approved for the treatment of PD motor symptoms, has been shown to reduce depressive behavior in rats and mice; these effects do not alter its efficacy on motor activity in any way, and therefore it was hypothesized that they were not dependent on the dopaminergic system [135]. A clinical trial in a cohort of 30 PD patients confirmed that istradefylline intake can improve depressive symptoms such as anhedonia and apathy [136].

Another type of non-motor symptom associated with PD is sleep disorders, which can manifest themselves as excessive daytime sleepiness as well as insomnia, fragmented sleep, night terrors, and hallucinations. These symptoms are made worse by dopamine replacement therapy [137]. Adenosine is known to be sleep-inducing through the actions of A_1_ARs and A_2A_ARs. However, the precise mechanisms underlying this action are still unknown, and the treatment of sleep disorders in PD patients remains difficult [138]. Preclinical studies on the effects of istradefylline and/or other A_1_ARs and A_2A_ARs ligands will be necessary to make up for this shortcoming. Thus far, two small clinical studies have been conducted on 21 and 14 PD patients, respectively. These studies found that treatment with istradefylline reduced daily sleepiness by increasing wakefulness without a negative impact on night sleep [139,140].

The data collected thus far therefore suggest that A_2A_ARs antagonists may be useful in the treatment of motor and non-motor symptoms, including depression and sleep disorders. Such compounds may also have positive effects on the cognitive deficits associated with the disease, particularly on short-term memory [141,142]. 

#### 2.3.2. Alzheimer’s Disease (AD)

AD is the most common cause of dementia, mainly affecting elderly individuals [143]. Due to the progressive aging of the population, this neurodegenerative disorder is on the rise, posing a significant health and societal burden. Amyloid plaques and neurofibrillary tangles are the two major pathological features of AD [144]. The amyloid cascade hypothesis suggests that neuronal death and synaptic dysfunction occur following alterations in amyloid β (Aβ) processing: the cleavage of amyloid precursor protein (APP) leads to the formation of Aβ peptides, which accumulate inside neuronal cells and extracellularly, wherein they aggregate into toxic plaques [144]. Neurofibrillary tangles are aggregates of hyperphosphorylated tau, a microtubule-associated protein that loses its affinity for microtubules and begins to self-assemble, disrupting neuron structure and function [145]. AD neuropathology is distinguished by the death of basal forebrain cholinergic neurons, resulting in reduced cholinergic transmission. From this characteristic derives the use of acetylcholinesterase inhibitors for the treatment of AD. The clinical use of muscarinic agonists as a different strategy to improve cholinergic transmission has been hampered by significant side effects [146]. Memantine, an NMDA receptor non-competitive antagonist, has been currently approved to treat the cognitive symptoms of AD [147].

In addition to cognitive decline, neuropsychiatric symptoms are common among patients suffering from AD. These include depression, psychosis, apathy, and aggression [148]. Furthermore, when compared to normal aging phase shifts, circadian sleep–wake cycles become accentuated [149]. Over the last few decades, advances in behavioral neuroscience and the neurocircuitry underlying brain functions have paved the way for the development of three major models that may account for neuropsychiatric symptoms in AD: the frontal-subcortical circuitry, cortico-cortical networks, and the ascending monoaminergic hypothesis [150].

The interest in adenosine and its receptors in AD stems from the observation that caffeine, a non-selective antagonist of ARs, improves human memory [151]. As a matter of fact, different studies support coffee’s favorable effects against cognitive decline and dementia, and caffeine intake may be associated with a decreased risk of AD [152]. The cognitive and neuroprotective effects of caffeine seem mainly related to the block of A_2A_ARs: in different experimental models of AD, A_2A_AR selective antagonists have a similar effect to caffeine [153,154]. One of the possible mechanisms underlying the neuroprotective action of A_2A_AR antagonists is the modulation of NMDA receptor functionality [155]. Furthermore, some studies have suggested that A_2A_AR activation may increase tau hyperphosphorylation, lending credence to the hypothesis that A_2A_AR antagonists have beneficial effects in AD [156,157]. The use of this strategy also seems justified by the overexpression of A_2A_AR in neurodegenerative diseases, including AD [158]. A_2A_AR density has been shown to be higher in the frontal cortex and hippocampus regions of AD patients, as well as aged or AD animal models [159,160,161].

Another important component of A_2A_AR signaling in AD is its control of neuroinflammation [162]. Some studies report a link between neuroinflammation and the neuropsychiatric symptoms of AD. A tumor necrosis factor (TNF)-alpha gene polymorphism has been associated both with AD and depression in the elderly [163]. In AD patients with depression, a statistical correlation between disease severity and serum cytokine levels has been found [164]. Moreover, the anti-inflammatory cytokine interleukin (IL)-10 showed reverse correlations with the total neuropsychiatric inventory score in patients with dementia, which manifested at the same time neuropsychiatric symptoms [165].

The importance of neuropathology in drug development is critical since the existing therapeutic options for neuropsychiatric symptoms may be less effective in the AD degenerating brain. Thus, understanding the dysfunction or dysregulation of the AD brain that generates neuropsychiatric symptoms will be required and could greatly advance the development of new strategies. In this context, A_2A_AR represents a good pharmacological target as it appears to be involved in various aspects concerning the pathogenesis of AD such as neurodegeneration and neuroinflammation, as well as being involved in the regulation of neurotransmission in brain areas important for behavior and mood.

## 3. Conclusions

Adenosine, interacting with its four receptor subtypes, is a subtle but important neuromodulator with multiple interconnections with numerous neurotransmitter systems. Through several preclinical studies and some clinical trials, researchers have tested the inhibitory or facilitating action of ARs as a potential therapeutic strategy for neuropsychiatric disorders. Nevertheless, the wide distribution of ARs and the numerous functions of adenosine in the body is considered a limiting factor for the drug development of AR ligands. Different factors must be taken into account for the future therapeutic application of AR interacting agents in neuropsychiatric disorders: brain barrier penetration, side effects, the complex pathogenetic mechanisms of these diseases, and the poor availability of reliable animal models. In conclusion, numerous efforts and further studies are required to exploit the huge therapeutic potential of the adenosinergic system in neuropsychiatric disorders.

## Figures and Tables

**Table 1:** Effect of A_1_ and A_2A_ARs in the CNS and therapeutic potential of their modulation in neuropsychiatric disorders.

Receptor Subtype	CNS Effects and Interactions	Pharmacological Strategy	Therapeutic Potential in Neuropsychiatric Diseases
A_1_ARs	Inhibition of neurotransmitter release Reduction of dopamine D_1_ signaling Reduction of neuronal excitability Increase of Homer1a expression	Activation	Depression Anxiety
A_2A_ARs	Reduction of dopamine D_2_ signaling Increase of excitatory neurotransmitter release Increase of mGLUR5 signaling Regulation of neuroinflammation	Activation	Schizophrenia Autism spectrum disorder
		Inhibition	Depression Fragile X syndrome Attention-deficit hyperactivity disorder Parkinson’s disease Alzheimer’s disease
